# Buffalo Academy of Medicine

**Published:** 1901-10

**Authors:** F. W. McGuire

**Affiliations:** Secretary


### Buffalo Academy of Medicine.

*Regular Stated Meeting, September 3, 1901.*

REPORTED BY F. W. McGUIRE, M. D., Secretary.

THE regular stated meeting of the Buffalo Academy of Medicine was held at the Academy rooms, Public Library Building, Tuesday, September 3, 1901.

The president, Dr. CHARLES G. STOCKTON called the meeting to order at 9 p.m.

The minutes of the last quarterly meeting were read and approved.

The council recommended the election of Drs. Henry E. Stadlinger, Edwin A. Bowerman and Frederick C. Busch for resident membership. They were separately balloted for and elected Fellows of the Academy.

The report of the Hartwig prize committee was presented, which is as follows:

"At the beginning of his term of office as President of the Buffalo Academy of Medicine for 1900-1901, Dr. Marcell Hartwig offered a prize of $50.00 for the best paper presented to the Academy by any one of the Fellows during his presidential term. The undersigned were appointed a committee to award the prize.

"Thirteen papers were presented to your committee. After careful examination and mature consideration the prize has been awarded to Dr. Irving Phillips Lyon for his paper entitled, Cancer distribution and statistics in Buffalo for the period 1880-1899, with reference to the parasitic theory."

Signed, J. W. GROSVENOR.
B. G. LONG.
S. Y. HOWELL.

On motion the report was received and filed and the committee discharged. Carried.

It was moved and seconded that the prize of $50.00 be presented by the President, Dr. Charles G. Stockton, to Dr. Irving P. Lyon, at the next stated meeting of the Academy. Carried.

On motion by Dr. Wende that the next meeting of the pathological section, September 17, be held at the University of Buffalo. Carried.

The section of medicine provided the following program: Intestinal dystrypsia, J. C. Hemmeter, Baltimore; Treatment of pneumonia, DeLancey Rochester. Doctor Hemmeter's paper was discussed by Drs. Benedict, Jones, Hartwig, Jewett and Stockton. Dr. Rochester s paper was discussed by Drs. Jones, Ullman, Thoma and Benedict. See report of medical section.

On motion by Dr. Thoma, a vote of thanks was tendered to Dr. Hemmeter for his very able and interesting discourse.

Attendance, 30; adjourned at 11 p.m.

---

### RYERSON'S SOLUTION.

| | | |
|---|---|---|
| ℞ | Sodii biborate | grs. xxx. |
| | Sodii bicarb. | grs. xxx. |
| | Sodii chlorid | grs. xxx. |
| | Sodii salicylas | grs. x |
| | Listerine | ℥i. |
| | Glycerinum pura | ℥i. |
| | Aq. dist. q. s. ad. | ℥viij. |

This is an excellent combination for nasal cleansing and disinfection. It is soothing, agreeable and effective. For shortness I call it Ryerson's solution.

*Extract from a medical article written by G. Sterling Ryerson, M. D., L. R. C. P. & S. etc., Toronto.*

